# Integrated transcriptomics, network pharmacology and clinical expression validation reveal the prognostic significance of PANoptosis-related genes in cordycepin-treated lung adenocarcinoma

**DOI:** 10.1007/s12672-026-05134-6

**Published:** 2026-06-08

**Authors:** Haoran Xu, Wenhui Huang, Qing Yang, Yuewei Chen, Peiyuan Su, Bing Fu

**Affiliations:** Hospital of Chengdu University of Traditional Chinese Medicine, Chengdu, 610072 Sichuan People’s Republic of China

**Keywords:** Cordycepin, PANoptosis, Lung adenocarcinoma, Prognosis

## Abstract

**Background:**

Studies have shown that PANoptosis is increasingly involved in cancer and cancer treatment. The Cordycepin has also been found to be involved in the development of various cancers. However, relevant research on both in lung adenocarcinoma (LUAD) remains relatively scarce. The present study aims to identify potential prognostic genes in LUAD and elucidate their impact on the prognosis of LUAD patients, with the goal of providing novel insights into the therapeutic strategies for this disease.

**Methods:**

The target genes for Cordycepin and transcriptomic datasets for LUAD were retrieved from public databases, and PANoptosis-related genes were retrieved from literature. This study employed differential expression analysis, consensus clustering analysis, weighted gene co-expression network analysis (WGCNA), and Cox regression analysis. This study identified potential biomarkers. The backpropagation neural networks (BPNNs) were constructed using these biomarkers. Furthermore, the study delved into underlying biological mechanisms through enrichment analysis. Additional analyses were performed to evaluate functional pathways, immune infiltration, and drug sensitivity in different risk individuals. Finally, clinical samples were collected and biomarker expression was validated using RT-qPCR.

**Results:**

A total of 8 biomarkers including SLC2A1, SMS, CCNA2, CDC25C, RNASE1, NR3C2, CAT, and ADA were identified in this study. Among these, SLC2A1, SMS, CCNA2, CDC25C, and ADA were significantly upregulated in clinical disease samples, while RNASE1, NR3C2, and CAT were significantly downregulated. The GSEA results indicated that the biomarkers were primarily enriched in mismatch repair and proteasome. There were 10 differential immune cells (Macrophages M0, Monocytes, activated dendritic cells, etc.) between two subgroups detected by the CIBERSORT algorithm. Meanwhile, 18 differential immune cells including Activated CD4+ T cells, Memory B cells, Natural killer T cells, etc. were identified using the ssGSEA algorithm. The analysis of drug sensitivity showed significant differences in 22 drugs between the two risk groups.

**Conclusion:**

Our results suggested that 8 biomarkers including SLC2A1, SMS, CCNA2, CDC25C, RNASE1, NR3C2, CAT, and ADA were associated with PANoptosis in LUAD. These findings may provide supportive evidence for the prognosis prediction and treatment of LUAD. However, the involvement of these genes in PANoptosis is currently only a hypothesis based on bioinformatics analysis and requires further experimental validation in the future.

## Introduction

Lung cancer (LC) is the leading cause of cancer-related mortality, among which the most common type of lung cancer is adenocarcinoma (LUAD), comprising approximately 40% of all lung cancer cases [1]. LUAD is a malignant epithelial tumor with glandular tubular differentiation or mucin secretion, originating mostly from bronchial mucosal epithelium, and is one of the most common types of lung cancer [2]. According to global cancer statistics, the incidence and mortality rates of lung cancer continue to rise, particularly among high-risk populations such as smokers and individuals with long-term exposure to environmental pollutants. This rising incidence and mortality underscores the growing public health significance of lung adenocarcinoma [3]. Despite the development of various therapeutic strategies for LUAD, such as tyrosine kinase inhibitors (TKIs) targeting the epidermal growth factor receptor (EGFR) and immunotherapies targeting programmed cell death protein 1 (PD-1) or programmed death-ligand 1 (PD-L1), its prognosis remains poor, with the 5-year overall survival rate of LUAD patients still around 16%. Therefore, understanding the molecular landscape of lung adenocarcinoma is crucial for promoting personalized medicine and targeted therapies. Notably, recent advances in genomics and molecular biology have identified key driver mutations and signaling pathways that fuel tumor growth, offering promising avenues for the development of targeted therapies. In view of this, there is an urgent need to identify potential prognostic genes within LUAD and understand their impact on the prognosis of LUAD patients, with the aim of providing new insights for the treatment of LUAD.

PANoptosis is a newly proposed programmed cell death (PCD) pathway, first put forward by American scholars Malireddi et al. in 2019. This pathway integrates key features of pyroptosis, apoptosis, and necroptosis, yet cannot be fully explained by these three PCD pathways alone [4]. Cordycepin is a derivative of adenosine extracted from Cordyceps militaris, which is utilized in traditional herbal supplements and alternative medicine for its diverse biological activities [5]. The general anticancer mechanism of cordycepin is mediated by regulation of the adenosine A3 receptor, the epidermal growth factor receptor (EGFR), mitogen-activated protein kinase (MAPK), and glycogen synthase kinase (GSK)-3β, leading to cell cycle arrest or apoptosis [6]. Studies have indicated that cordycepin can inhibit the migration and invasion of human liver cancer cells by downregulating the expression of CXCR4. Additionally, it can suppress the migration and invasion of triple-negative breast cancer cells by modulating epithelial-mesenchymal transition transcription factors (EMT-TFs) such as SLUG, TWIST1, SNAIL1, and ZEB1. Furthermore, cordycepin hinders the development of colon cancer through the gsk3β/β-catenin/cyclin D1 signaling pathway [7]. These findings underscore the potential of cordycepin as a potent anticancer agent, showcasing its diverse regulatory effects on cellular behavior across different cancer types.

In recent years, the rapid advancement of bioinformatics has greatly advanced an in-depth understanding of the occurrence and progression of diseases. Researchers can not only utilize ever-updating bioinformatics tools but also combine newly developed experimental methods to validate these bioinformatics-driven hypotheses. When bioinformatics and experimental tools are effectively integrated, they enable researchers to explore the complexity of diseases. Meanwhile, the rise of network pharmacology (NP) has provided a novel perspective for deciphering and visualizing the potential interaction networks of complex drugs with multifactorial diseases. The continuous development and application of network pharmacology have improved the reliability of drug development and therapeutic efficacy, particularly demonstrating tremendous potential in multi-target drug design and the treatment of complex diseases. In the field of lung cancer [3], bioinformatics and network pharmacology have also been widely applied. For instance, Junsha An and colleagues identified potential hub genes and their biological mechanisms in rheumatoid arthritis and non-small cell lung cancer through bioinformatics analysis, providing a new theoretical basis for the early diagnosis and targeted therapy of lung cancer [8]. Furthermore, Xiaocong Liu and colleagues, through network pharmacology studies, found that Psidium guajava leaf can regulate multiple signaling pathways in tumor cells, revealing new directions for drug development and providing potential novel strategies for cancer treatment [9]. In summary, the integration of bioinformatics and network pharmacology not only profoundly promotes the understanding of disease mechanisms but also offers brand-new ideas for drug development and disease treatment. Particularly in the research of complex diseases such as tumors, it has shown great promise for application.

This study employing bioinformatics techniques combined with network pharmacology to identify molecular subtypes associated with Panoptosis-Related Genes (PRG) in LUAD. This study further evaluates the prognostic value and tumor microenvironment of these subtypes, and constructs and validates a prognostic model based on PRGs. The objective is to provide valuable insights and support for the prognosis and treatment of LUAD.

## Materials and methods

### Data sources

TCGA-LUAD dataset (including clinical and survival information) were achieved in UCSC database (https://xenabrowser.net), containing 526 Tumor and 59 Normal samples. GSE68465 (442 Tumor and 20 Normal samples) and GSE31210 (226 Tumor samples and 20 Normal samples) datasets were achieved from GEO database (http://www.ncbi.nlm.nih.gov/geo/). GSE68465 dataset was utilized for validation of the risk model, and the GSE31210 dataset was utilized as analysis of gene expression. Moreover, a total of 347 PRGs were collected from the relevant literature (Supplementary Table 1) [10].

Target genes for cordycepin were retrieved from six public databases, including TCMSP (https://old.tcmsp-e.com/tcmsp.php), CTD (http://ctdbase.org/), SEA (https://sea.bkslab.org/), SuperPred (https://prediction.charite.de/), SymMap (http://www.symmap.org/) and HERB (http://herb.ac.cn). Among these, CTD, HERB and SymMap provide gene names directly; SEA provides data in CSV format; after splitting the data using the separate function in the R package tidyr (version 1.3.1), human targets were filtered and GAPDH was removed; SuperPred provides UniProt IDs, which were converted to official gene symbols using the bitr function in the R package clusterProfiler (version 4.7.1.3); TCMSP provides protein names, which are first matched to UniProt IDs via the UniProt database and then further converted to official gene symbols. The gene lists processed from the above six databases were merged, and the distinct function in the R package dplyr (version 1.1.4) was used to perform exact deduplication based on official gene symbols, ultimately yielding 480 cordycepin target genes (CTGs).

### Subtype determination

Firstly, PRGs were subjected to univariate COX analysis to screen out the PRGs related to prognosis (*p* < 0.001), and these genes were utilized for subtype identification. Then, based on the prognosis-related PRGs, the consistency clustering analysis was implemented on samples in TCGA-LUAD dataset using the ConsensusClusterPlus (v 1.58.0) package [11], and the best clustering method was selected. The clustering results were evaluated by principal component analysis (PCA). For further comparing the survival differences between different LUAD subtypes, the Kaplan–Meier (K–M) curves were plotted by the survival (v 3.3-1) package [12].

### Identification and protein-protein interactions (PPI) analysis of overlapping genes

In our study, differentially expressed genes between LUAD and Normal samples (DEG1), and between different subtypes (DEG2) were acquired via the DESeq2 (v 1.34.0) [13] package (|Log2FC| > 0.5, p.adj < 0.05). The pheatmap (v 1.0.12) [14] and ggplot2 (v 3.3.5) [15] packages were applied for visualization. Then, WGCNA was conducted on 515 LUAD samples in training set. Outlier samples were excluded via sample clustering. A soft threshold (β) was determined to construct a scale-free network. Genes were divided into distinct modules. The subtypes of LUAD were used as traits, and association between the modules and traits was assessed by Pearson method. The module that most associated with trait was selected as critical module.

CTGs, DEGs1, DEGs2, and critical module genes were intersected to obtain overlapping genes. Furthermore, a PPI network of those genes was developed via the STRING database (https://cn.string-db.org/) to forecast the interactions among overlapping genes (confidence = 0.3).

### Development and validation of prognostic model

Firstly, overlapping genes were subjected to univariate Cox regression analysis to recognize prognosis-related candidate genes (*p* < 0.05). Subsequently, Least absolute shrinkage and selection operator (LASSO) regression analysis was performed on the candidate genes using the R package glmnet (v 4.1-4) [16]. Parameter tuning was carried out via 10-fold cross-validation, with the objective of minimising partial likelihood bias. The λ value corresponding to the minimum cross-validation error (lambda.min) was selected as the optimal regularisation parameter, and genes with non-zero regression coefficients were ultimately identified as biomarkers. Next, a risk model of biomarkers was developed. According to the median risk score (risk score = $\:\sum\:_{1}^{\mathrm{n}}\mathrm{c}\mathrm{o}\mathrm{e}\mathrm{f}\left(\mathrm{g}\mathrm{e}\mathrm{n}\mathrm{e}\mathrm{i}\right)\mathrm{*}expression\left(\mathrm{g}\mathrm{e}\mathrm{n}\mathrm{e}\mathrm{i}\right)$). Then, LUAD patients in training set and GSE68465 validation set were classified into the high- and low-risk groups. K-M survival curves and receiver operating characteristic (ROC) curves (1-, 2- and 3-year) were plotted for patients in both datasets.

### Nomogram establishment and validation

For obtaining independent prognostic factors of LUAD patients, combining with risk score and clinical features (Tumor Stage, Smoking status, Gender, Pathological M, Pathological N, Age, and Pathological T), a nomogram model for clinical survival prediction was created. Firstly, Univariate Cox regression analysis was applied on risk score and above clinical features. Then, the PH hypothesis test was carried out, to select the clinical features suitable for creating the multivariate Cox model. Based on the retained factors, a nomogram for predicting survival rates of LUAD patients (1-, 2- and 3-year) was developed. K-M survival analysis was performed. ROC and calibration curves were applied to verify the model validity.

### Correlation analysis

Furthermore, to examine the differences of clinical characteristics between risk subgroups, the heat map was utilized to show the distribution of biomarkers across clinical features. Wilcoxon test method was utilized to analyze the risk score distribution differences.

Moreover, LUAD patients were grouped based on differential clinical features, and K-M survival analysis was conducted on different subgroups of these differential clinical features.

### Immune microenvironment analysis

For examining LUAD immune infiltration profiles, CIBERSORT and ssGSEA algorithms were performed to estimate immune cell infiltration level between risk subgroups, respectively. Moreover, this study further analysed the differences in immune cell infiltration between the two subgroups and calculated the Spearman’s correlation coefficient between the infiltration scores of the different immune cell types and the risk scores (cor > 0.3, *p* < 0.05). Expression differences of 19 immune checkpoints between subgroups were evaluated. In addition, the ESTIMATE algorithm was conducted to compute immune, stromal, tumor purity and ESTIMATE scores. Differences of those scores between two subgroups were analyzed.

### Drug sensitivity analysis

For obtaining clinical therapeutic drugs for LUAD, 538 chemotherapy drugs for LUAD were achieved using the Genomics of Drug Sensitivity in Cancer (GDSC) online database (https://www.cancerrxgene.org/). Moreover, 538 drugs in GDSC online database and 138 common chemotherapy and molecularly targeting drugs acquired by pRRophetic (v 0.5) package [17] were crossed to acquire the common drugs (co-drugs). Next, the IC50 of each co-drug in each LUAD sample in training set was computed. Wilcoxon test method was utilized to evaluate the IC50 of chemical drugs with remarkable sensitivity differences between risk subgroups. Finally, associations between the IC50 of chemical drugs and risk scores were analyzed.

### Functional analysis of biomarkers

For investigating biomarker-related functional pathways in LUAD, moreover, ‘c2.cp.kegg.v7.5.1.symbols.gmt’ was selected as a reference gene set, and Gene Set Enrichment Analysis (GSEA) was subsequently carried out via clusterProfiler (v 4.0.5) package [18]. The selected biomarkers and PANoptosis subtype genes were imported into the STRING database, with a confidence threshold set at 0.4, to identify high-confidence protein-protein interactions. The interaction data were imported into Cytoscape, where node connectivity was calculated to identify the most highly connected hub genes, followed by Reactome pathway enrichment analysis (*p* < 0.05).

### Expression validation

Biomarker expressions were further analyzed. Biomarker expression trends in LUAD and Normal groups within training and GSE31210 validation sets were assessed by Wilcoxon test method (*p* < 0.05). Meanwhile, tumor samples from 5 LUAD patients and 5 adjacent normal tissue samples were collected in Hospital of Chengdu University of Traditional Chinese Medicine for reverse transcription-quantitative PCR (RT-qPCR) analysis to examine differential biomarker expression in clinical samples. Specifically, total RNA was isolated by TRIzol reagent (Vazyme, China) and reverse-transcribed into cDNA via Hifair^®^ III 1st Strand cDNA Synthesis SuperMix for qPCR kit (Yeasen, China). Subsequently, primer sequences for the biomarkers and the internal reference gene GAPDH were designed (Table 1). After diluting cDNA products, PCR amplification was performed, and a 40-cycle reaction was carried out on a CFX Connect real-time quantitative fluorescence PCR instrument (BIO - RAD, USA) (Table 2). Finally, each sample was analysed in triplicate; relative expression levels were calculated using the 2−△△CT method, and Graphpad Prism 10 was used for visualization and difference comparison (*p* < 0.05).

**Table 1:** Primer sequence

Primer	Sequence
SLC2A1 F	TCTCGAAACTGGGCAAGTCC
SLC2A1 R	TCACCCACATACATGGGCAC
SMS F	AAGAACGGCAGATTACCACCC
SMS R	TCTTCTTTGCCACTGCCCAT
CCNA2 F	CTGGTGGTCTGTGTTCTGTGA
CCNA2 R	TCTTGGATGCCAGTCTTACTC
CDC25C F	ATGACAATGGAAACTTGGTGGAC
CDC25C R	GGAGCGATATAGGCCACTTCTG
RNASE1 F	CTGCAGATCCAGGTCACTTTG
RNASE1 R	GGAGCCGGACAAGAGACTTC
NR3C2 F	GTCAGTCACCTGGGTGCAAG
NR3C2 R	GCTCCACAGCCTGAGAAACT
CAT F	ACTTCTGGAGCCTACGTCCT
CAT R	AAAGTCTCGCCGCATCTTCA
ADA F	GGAACCAGGCTGAACTGGTC
ADA R	TGGTCTTCCAGGGTGTGGTA
GAPDH F	ATGGGCAGCCGTTAGGAAAG
GAPDH R	AGGAAAAGCATCACCCGGAG

**Table 2:** PCR program

	Temperature (°C)	Time
Pre-denaturation	95	1 min
Denaturation	95	20 s
Annealing	55	20 s
Extension	72	30 s

### Statistical analysis

Analyses in our study were implemented in R (v 4.1.0) package and the related public databases.

## Results

### Two subtypes of LUAD were obtained

**Fig. 1:**
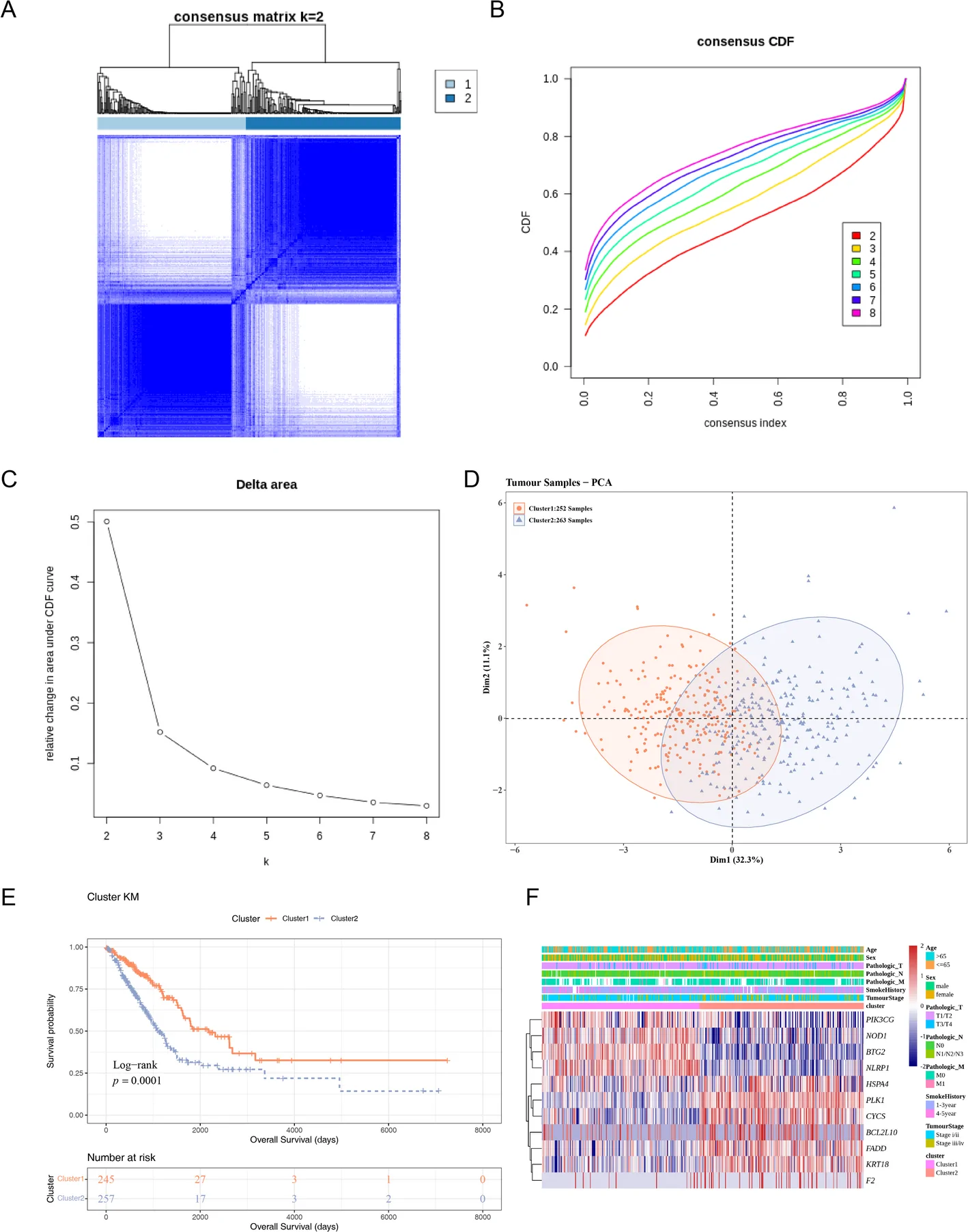
Two subtypes of LUAD were obtained. **A** Unsupervised clustering diagram. **B** Consensus cumulative distribution function (CDF) plot. The CDF curve helps determine the optimal number of clusters by identifying the k at which the CDF reaches a plateau. **C** Delta area plot showing the relative change in area under the CDF curve as a function of the number of clusters (k). The delta area helps identify the optimal number of clusters by observing the point where the curve plateaus. **D** Results of the PCA analysis for subtypes. Orange represents the first cluster of the PANoptosis subtype, whilst blue represents the other cluster. The fact that samples within each cluster are clustered together indicates a high degree of similarity between these samples and good intra-cluster consistency. The areas where the two clusters overlap indicate a certain degree of correlation between the groups; the smaller the overlap, the better. The figure here demonstrates that the two subtypes can be clearly distinguished. **E** KM curves for different subtypes. The x-axis represents survival time, and the y-axis represents survival rate. **F** Heatmap showing the expression levels of prognosis-related PRGs across different clinical characteristics in samples from the two subtypes

A total of 11 PRGs related to prognosis including KRT18, PLK1, HSPA4, BCL2L10, BTG2, FADD, NLRP1, F2, PIK3CG, NOD1, and CYCS were recognized via univariate Cox regression analysis. Moreover, consistency clustering analysis showed that it was suitable to cluster LUAD into 2 subtypes (K = 2) (Fig. 1A–C). The PCA results indicated that the two subtypes could be well distinguished (Fig. 1D). Meanwhile, there were significant survival differences between LUAD patients of different subtypes, and the Cluster 1 subtype exhibited a higher survival possibility (Fig. 1E). Expression levels of PRGs related to prognosis in different clinical features of the two subgroups of samples were shown in Fig. 1F. Among these, PIK3CG, NOD1, BTG2 and NLRP1 showed low expression in cluster 2, whilst the remaining seven genes exhibited high expression. Furthermore, in this dataset, the proportion of T1/T2-stage cases was higher than that of T3/T4-stage cases, and the proportion of stage I/II patients was also greater than that of stage III/IV patients.

### Recognition of DEGs and critical modules

**Fig. 2:**
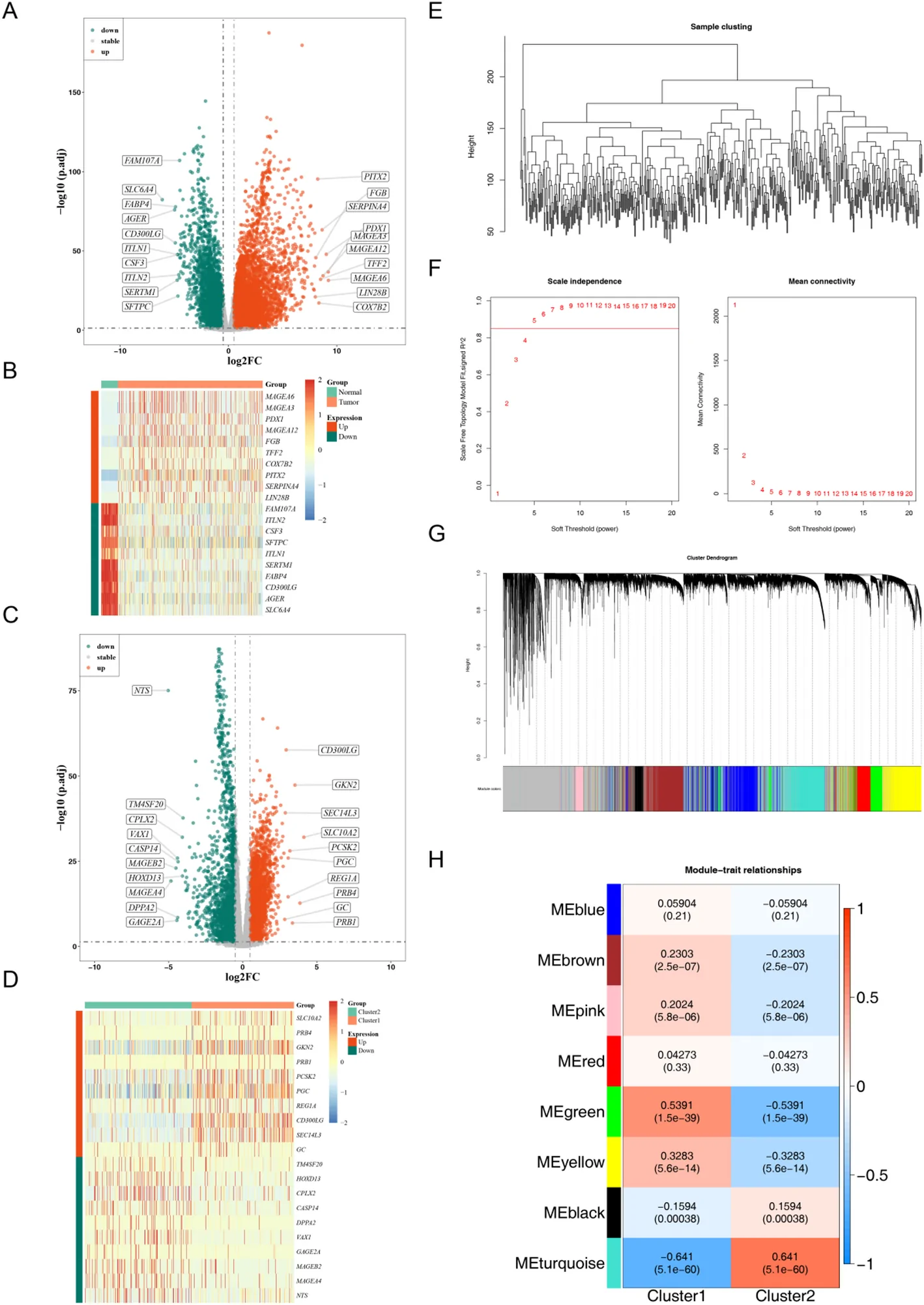
Identification of differentially expressed genes and key modules in LUAD. **A** A volcano plot showing genes differentially expressed between LUAD tissue and normal tissue. The x-axis represents LogFC values, and the y-axis represents − log10(p.adj). Red dots represent up-regulated genes, green dots represent down-regulated genes, and grey dots represent genes with non-significant expression. **B** Heatmap of differentially expressed genes between LUAD and normal tissues. Colour represents the relative expression level of the gene, with red indicating relatively high expression and blue indicating relatively low expression. The top 10 up-regulated and 10 down-regulated genes based on logFC are displayed. **C** Volcano plot of differentially expressed genes between the two subtypes. **D** Heatmap of differentially expressed genes between the two subtypes. **E** Construct a hierarchical clustering tree to detect outliers. **F** Scale-free fitting indices and average connectivity with a soft threshold (β). **G** Cluster Dendrogram of Consensus Clustering. This dendrogram illustrates the results of sample-level clustering based on the consensus matrix; the coloured bars below represent the different modules. **H** Heatmap showing the correlation between gene modules and traits. The x-axis shows grouping information, whilst the y-axis represents different modules

In total, 9484 DEGs1 between LUAD samples and Normal samples were acquired (Fig. 2A, B, Supplementary Table 2). A total of 5032 DEGs2 between Cluster 1 and Cluster 2 of LUAD were detected (Fig. 2C, D, Supplementary Table 3). After confirming that there were no outlier samples, WGCNA was conducted (Fig. 2E). After setting soft threshold of 5, a scale-free network was established (Fig. 2F). Eight trait-associated modules were identified (Fig. 2G). The turquoise module showed the strongest association with traits, leading to its identification as critical module (|Cor| = 0.641, *p* < 0.05). Following this, 2649 genes in this module were retained for further analysis (Fig. 2H).

### Protein-level interactions of 31 overlapping genes

**Fig. 3:**
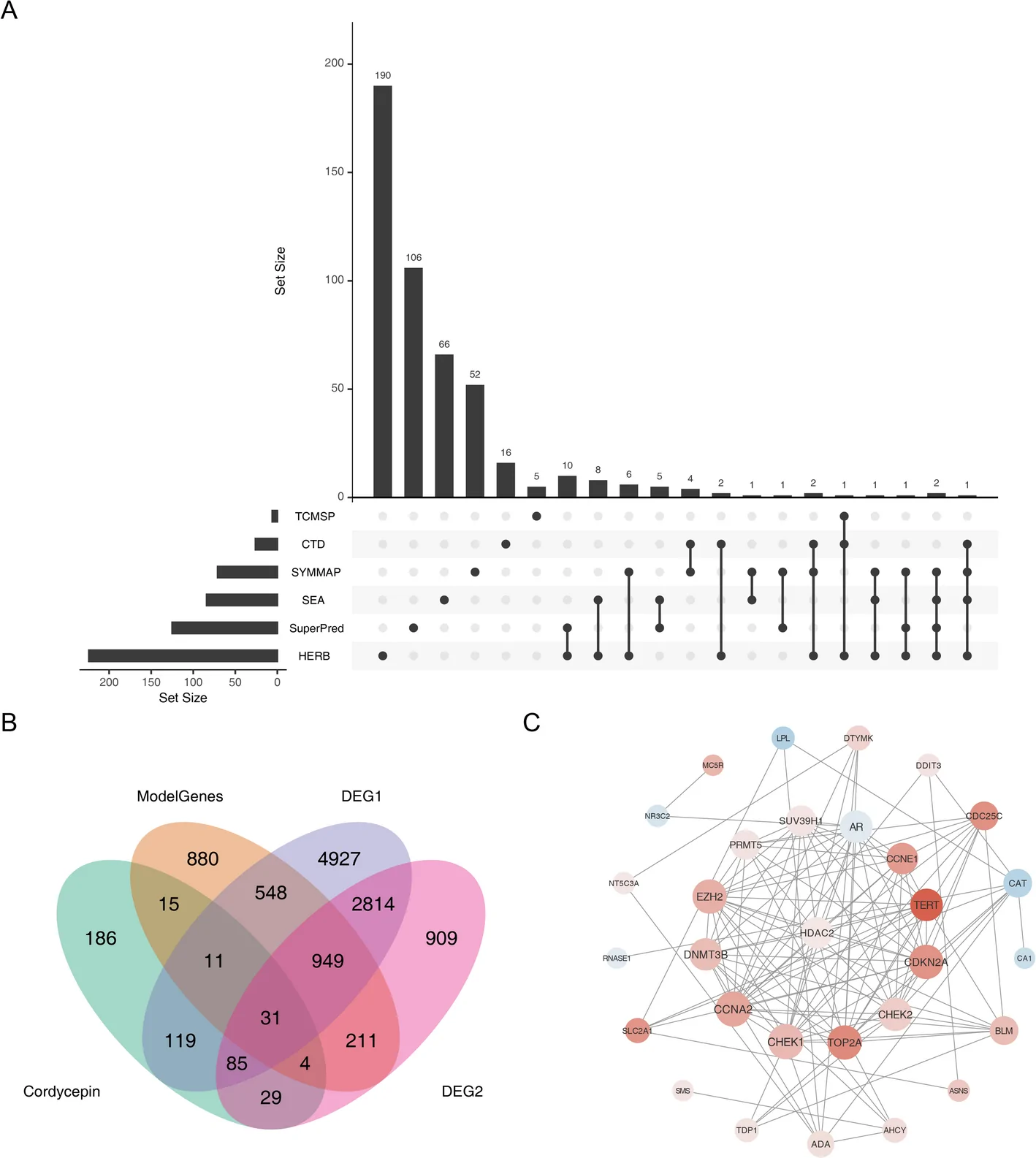
Overlapping genes and PPI network. **A** Merge six databases to identify target genes associated with cordycepin. **B** Generate a Venn diagram showing 31 overlapping genes. **C** Protein–protein interaction network of the overlapping genes. Nodes represent the overlapping genes; colours correspond to the logFC values of disease-associated differentially expressed genes, with red indicating upregulation and blue indicating downregulation. The intensity of the colour reflects the magnitude of the value, whilst the size of the node indicates the degree of connectivity

After, a merged and deduplicated analysis was conducted among the results from 6 databases, 480 CTGs were achieved (Fig. 3A). According to the intersection of 480 CTGs, 9484 DEGs1, 5032 DEGs2, and 2649 module genes, 31 overlapping genes were identified (Fig. 3B). Besides, a PPI network with 30 nodes and 122 edges was generated, elucidating the complex interplay of overlapping genes at the protein level. Remarkably, HDAC2 showed a higher connectivity (Fig. 3C).

### Identification of SLC2A1, SMS, CCNA2, CDC25C, RNASE1, NR3C2, CAT, and ADA as biomarkers

**Fig. 4:**
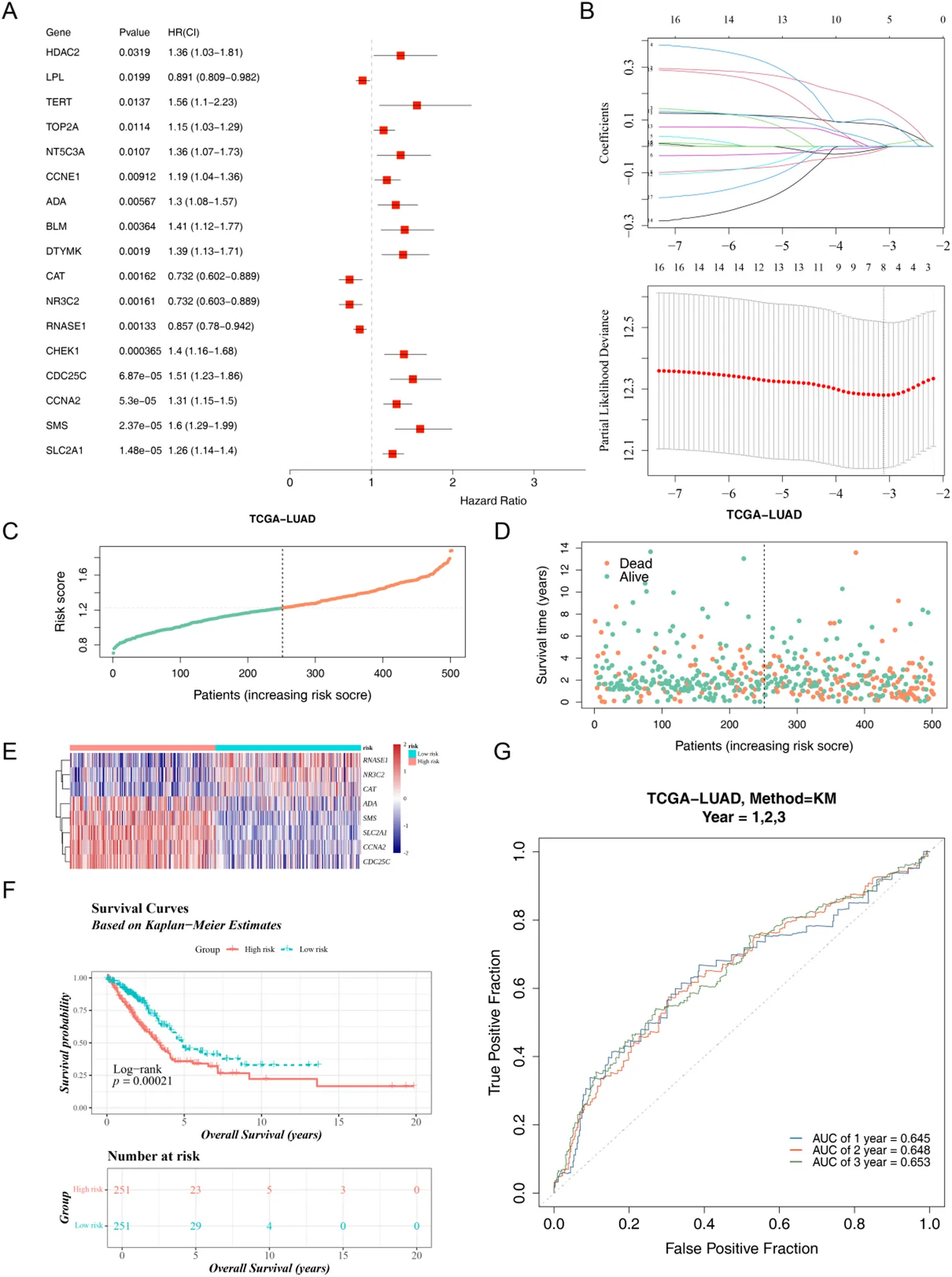
Identification of eight biomarkers and construction of a risk model for LUAD. **A** Forest plot of a univariate COX analysis. **B** LASSO regression cross-validation plot and profile of genes with LASSO coefficients. **C** Training set risk scores. The x-axis shows patient samples sorted by risk score, with scores increasing from left to right; the y-axis shows the risk scores; the horizontal dotted line indicates the median risk score; the vertical dotted line indicates the sample corresponding to the median risk score. **D** Survival distribution of LUAD patients (TCGA-LUAD). The x-axis represents the patient sample ranked by risk score, with risk scores increasing from left to right; the y-axis represents patient survival time, and the dotted line indicates the number of patients corresponding to the median risk score. **E** Heatmaps showing the expression of eight biomarkers in high- and low-risk groups (TCGA-LUAD). The colours represent the relative expression levels of the genes, with red indicating relatively high expression and blue indicating relatively low expression. **F** Kaplan-Meier analysis comparing high- and low-risk groups (TCGA-LUAD). The upper section shows survival time and survival probability curves, with the x-axis representing the total survival time of the sample and the y-axis representing the survival probability. The lower section presents a risk table showing the number of subjects remaining in each group at different survival times. **G** Time-dependent ROC curve (TCGA-LUAD). The x-axis represents the false positive rate; the y-axis represents the true positive rate

There were 17 prognosis-related candidate genes screened via univariate Cox regression analysis (Fig. 4A). Under the optimal model parameter λ = 0.04441359 (corresponding to the minimum mean cross-validation error), the LASSO algorithm identified a total of eight biomarkers: SLC2A1, SMS, CCNA2, CDC25C, RNASE1, NR3C2, CAT and ADA (Fig. 4B). A risk model was constructed based on the aforementioned eight genes, and patients were classified into high-risk (*n* = 251) and low-risk (*n* = 251) groups using the median risk score as the threshold (Fig. 4C). As the risk score increased, the number of patient deaths also showed an increasing trend (Figs. 4D). The heatmap shows that SLC2A1, NR3C2 and CAT were highly expressed in the low-risk group, whilst the remaining genes were lowly expressed in the low-risk group (Fig. 4E). Furthermore, a remarkable difference in prognosis of LUAD patients between these two risk subgroups was observed (*p* < 0.05). Patient prognosis was better in low-risk group (Fig. 4F). In training set, the AUC values for the risk model’s predictions of patient survival status at years 1, 2 and 3 were all above 0.6, demonstrating good survival prediction performance. (Fig. 4G). Besides, in GSE68465 validation set, LUAD patients were classified into high- (*n* = 221) and low-risk (*n* = 221) groups. The associated results are similar to those of the training set (Supplementary Fig. 1A–E). Consequently, the generalizability of this risk model was superior, and it might be a valuable tool for risk assessment in clinical practice for LUAD.

### Development of a nomogram with robust predictive performance

**Fig. 5:**
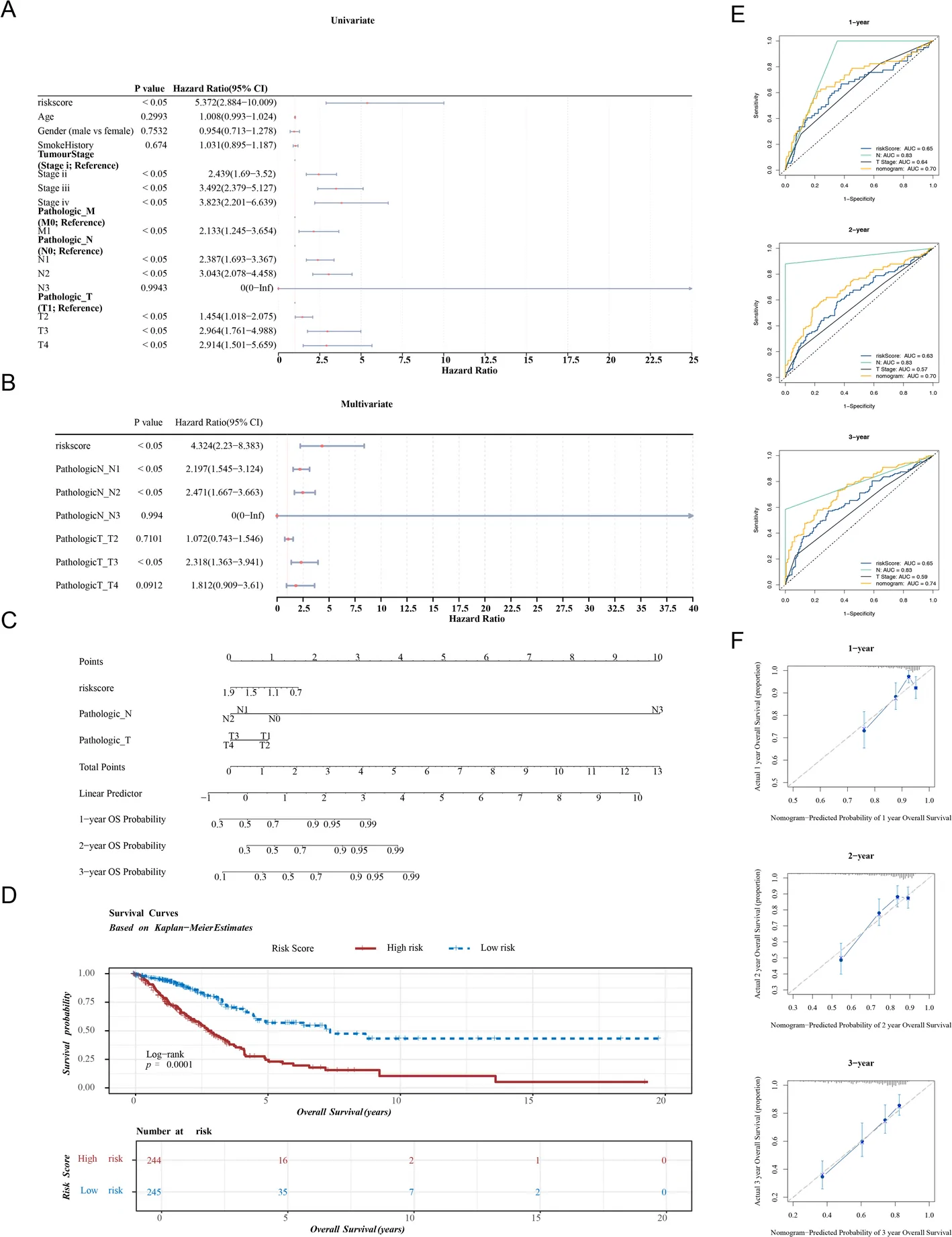
Development of a nomogram with robust predictive performance. **A** Identify clinical indicators associated with prognosis using univariate Cox regression analysis. **B** Forest plot for multivariate Cox analysis. **C** Nomograms for predicting the 1-year, 2-year and 3-year prognosis in LUAD patients. The first section, “Points”, indicates the individual score for each variable when the risk score is a specific value; the second section, “Variables”, shows the range of values for the line segment following each variable, representing the variable’s total contribution to the outcome event, whilst the scale marks on the line segment indicate the different values of the variable; the third section, “Total Points”, represents the sum of the individual scores for the variable values; the fourth section, “1–3-year survival probability predictions”, indicates the survival probability derived from the patient’s total score after conversion. **D** Based on the line chart, the overall risk score was calculated, and the training cohort was divided into two groups according to the median for Kaplan-Meier analysis. The x-axis represents survival time (years), and the y-axis represents the probability of survival. **E** 1-, 2- and 3-year ROC analyses of the nomogram. **F** 1-, 2- and 3-year calibration curves of the nomogram. The x-axis represents the probability of survival predicted by the nomogram, and the y-axis represents the actual probability of survival

After univariate Cox analysis and PH hypothesis test, risk score, Pathologic T, and Pathologic N were retained to create the multivariate Cox model (Fig. 5A, B). Based on these factors related to survival and prognosis of LUAD patients, a nomogram was established for survival prediction (1-, 2- and 3-year) (Fig. 5C). Significant survival differences between two subgroups were observed in K-M survival curves (Fig. 5D). The ROC curve analysis of the nomogram showed that the AUC values for predicting patient survival status at 1, 2, and 3 years were all greater than 0.7, indicating good discriminative ability (Fig. 5E). Additionally, the calibration curve demonstrated that the predicted survival probabilities were close to the ideal reference line, suggesting that the nomogram had favorable predictive accuracy (Fig. 5F). Summarily, these results highlighted the remarkable clinical applicability of risk score, and this nomogram model could serve as an effective tool in the management of LUAD.

### Association between clinical features and risk scores

**Fig. 6:**
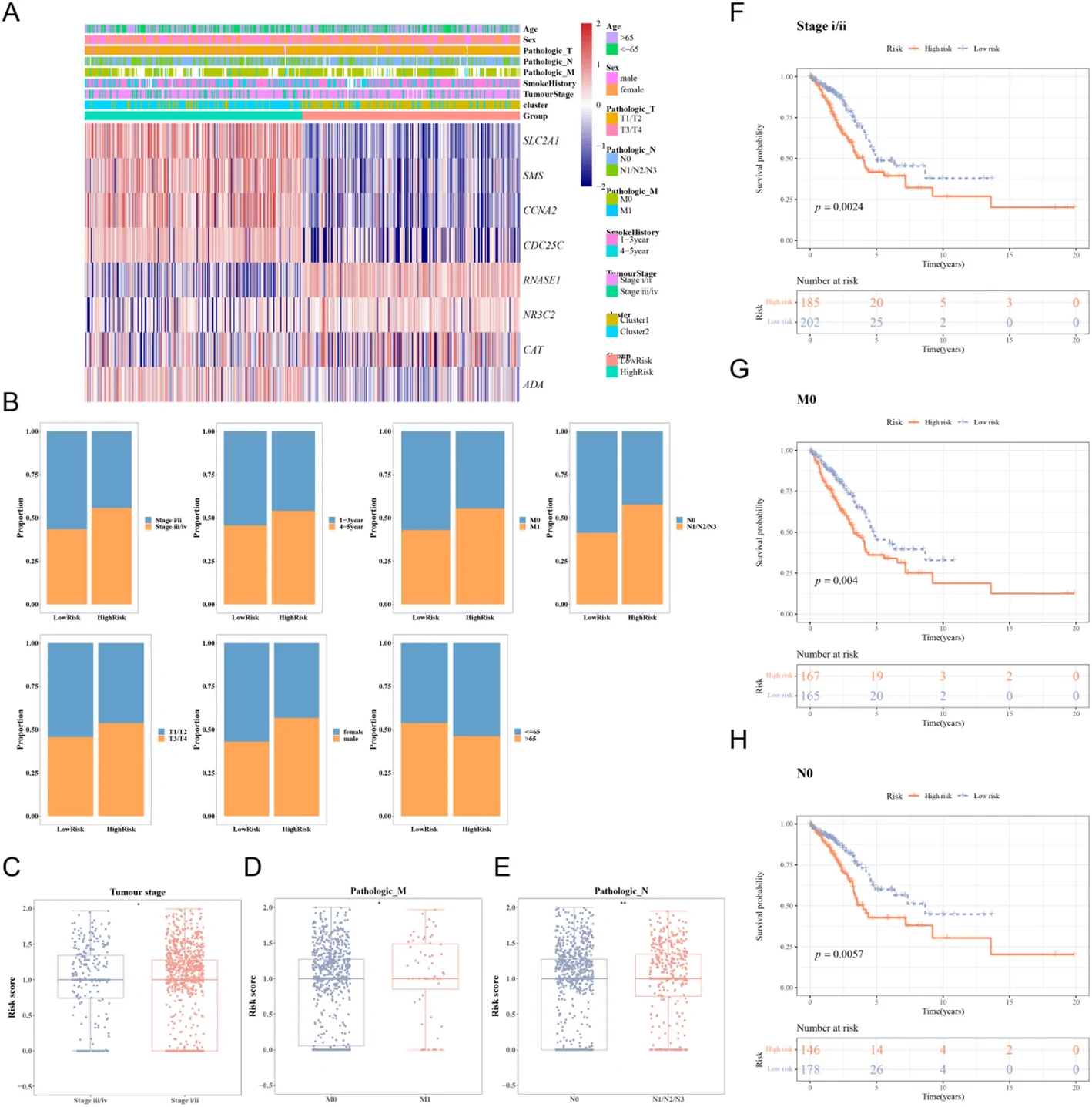
Association between clinical features and risk scores. **A** Heatmap showing the distribution of biomarkers across different clinical characteristics. **B** Distribution of different clinical characteristics across high- and low-risk groups. **C**–**E** Differences in different clinical characteristics between high- and low-risk groups. * *p* < 0.05, ** *p* < 0.01; ns: not significant. (F-H) Patients were stratified into subgroups based on clinically significant differences, and Kaplan–Meier survival analyses were conducted within each subgroup

The samples were divided into two subgroups, Cluster 1 and Cluster 2, following cluster analysis, which largely corresponded to the high- and low-risk groups (Fig. 6A). The number of LUAD patients with clinical features including Stage ⅲ/ⅳ, 4–5 year, Pathologic M1, Pathologic N1/N2/N3, Pathologic T3/T4, and male highly distributed in high-risk group (Fig. 6B). Risk score was remarkable difference between patients with different clinical characteristics, including Tumor stage, Pathologic M and Pathologic N (Fig. 6C–E). Moreover, we could find a better prognosis in low-risk group with Tumor stage i/ii, Pathologic M0 and Pathologic N0 (Fig. 6F–H). These findings indicated that individuals with higher risk scores might exhibit greater disease severity and potentially poorer prognosis.

### Immune microenvironment characteristics in LUAD

**Fig. 7:**
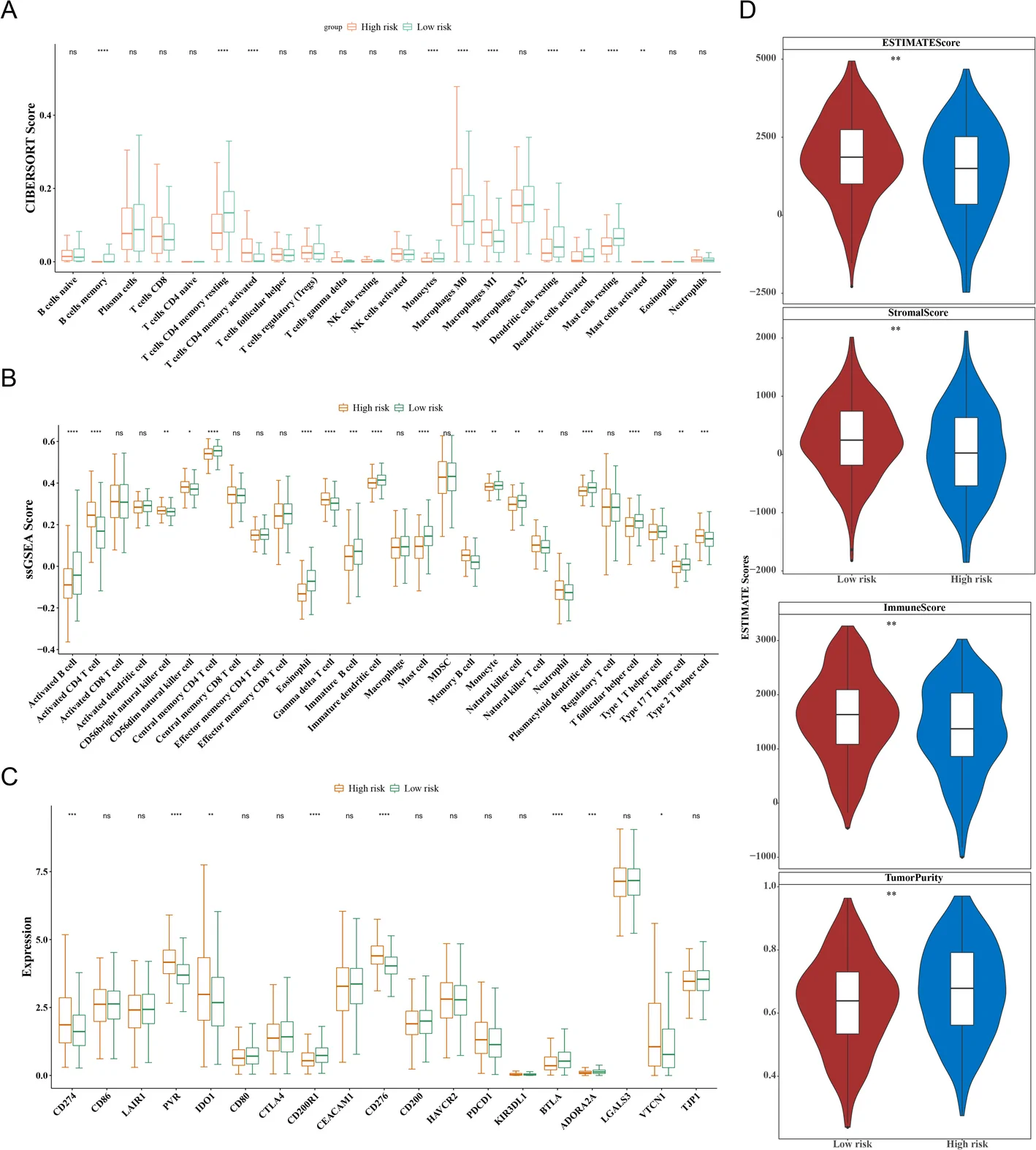
Immune microenvironment characteristics in LUAD. **A** CIBERSORT immune infiltration analysis. Expression of 22 immune cell types in the low-risk and high-risk groups. ** *p* < 0.01, **** *p* < 0.0001, ns: not significant. **B** ssGSEA immune infiltration analysis. Expression of 28 immune cell types in the low-risk and high-risk groups. * *p* < 0.05, ** *p* < 0.01, *** *p* < 0.001, **** *p* < 0.0001; ns: not significant. **C** Immune checkpoint expression analysis. Analysis of the expression of 19 immune checkpoints between the low-risk and high-risk groups. * *p* < 0.05, ** *p* < 0.01, *** *p* < 0.001, **** *p* < 0.0001; ns: not significant. **D** ESTIMATE algorithm calculation of immune, stromal and ESTIMATE scores between the low-risk and high-risk groups. ** *p* < 0.01

There were 10 differential immune cells (Macrophages M0, Monocytes, Dendritic activated cells, etc.) between two subgroups by the CIBERSORT algorithm (p.adj < 0.05) (Fig. 7A). Meanwhile, 18 differential immune cells like Activated CD4 T cell, Natural killer T cell, Memory B cell and so on between subgroups were identified using the ssGSEA algorithm (p.adj < 0.05) (Fig. 7B). In particular, activated CD4 T cells and memory B cells showed a significant positive correlation with the risk score, meaning that their infiltration levels increased as the risk score rose; conversely, eosinophils and mast cells showed a significant negative correlation with the risk score, with their infiltration levels decreasing as the risk score rose (Supplementary Fig. 2). We found 8 immune checkpoints including BTLA, PVR, CD274, CD276, CD200R1, VTCN1, ADORA2A, and IDO1 were markedly expressed in two risk subgroups (p.adj < 0.05) (Fig. 7C). The immune, stromal, and ESTIMATE scores were markedly elevated in low-risk group, while tumor purity score was markedly lower (Fig. 7D). Consequently, risk score might affect the prognosis of LUAD by altering microenvironment characteristics.

### Potential of risk score as predictor of drug treatment outcomes

**Fig. 8:**
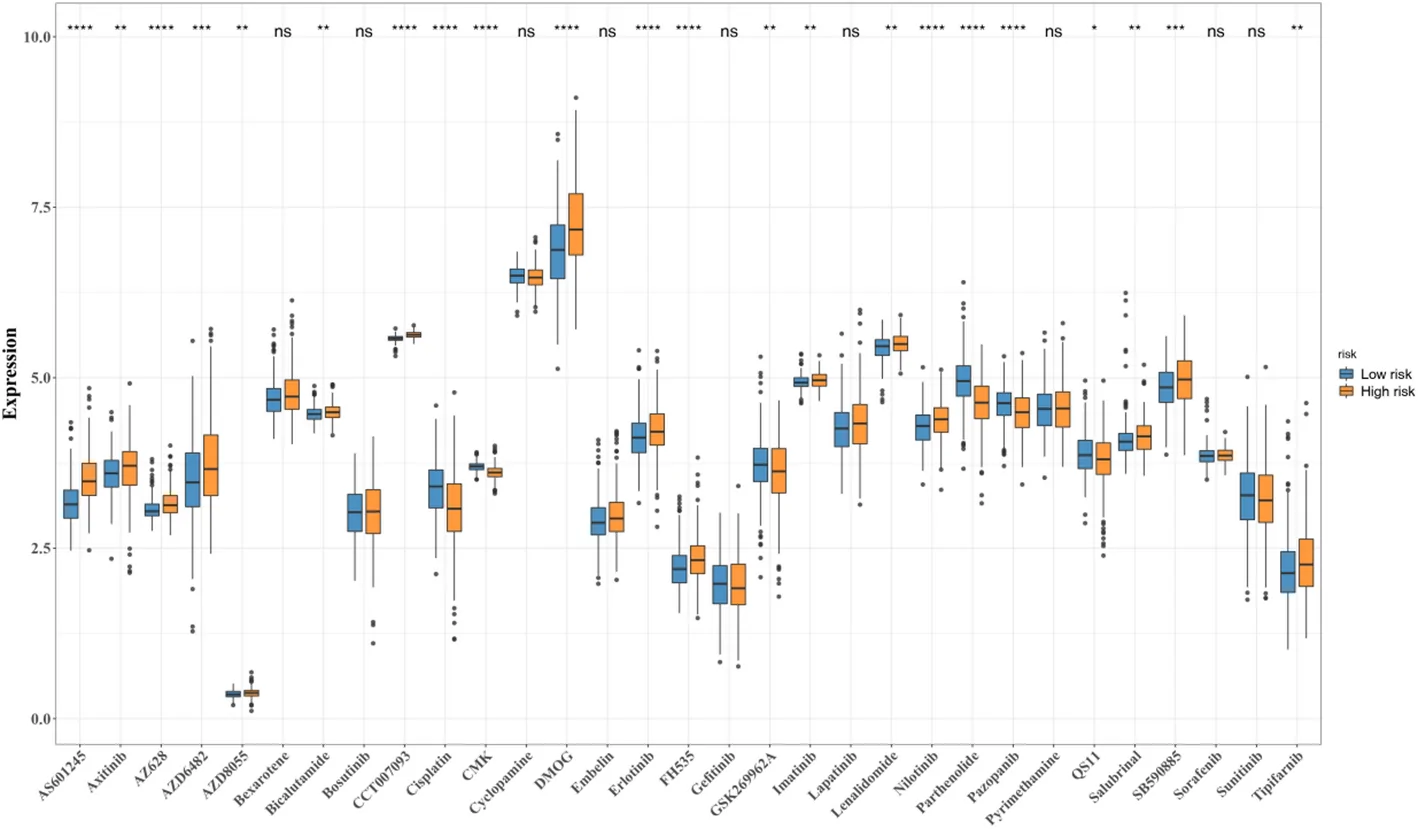
Differences in the IC₅₀ values of 31 drugs between the low-risk and high-risk groups. * *p* < 0.05, ** *p* < 0.01, *** *p* < 0.001, **** *p* < 0.0001; ns: not significant

Furthermore, analysis of drug sensitivity revealed significant differences in IC50 values across 2 risk groups for a total of 22 drugs (including AS601245, AZ628, AZD6482, etc.) (Fig. 8). Risk score was positively associated with three drugs (AS601245, CCT007093, and DMOG) (Cor > 0.3). Moreover, risk score was inversely linked to three drugs (Cisplatin, CMK, and Parthenolide) (Cor < -0.3). It was noted that the above results were derived from computational predictions and had not yet been validated by in vitro or in vivo experiments. Therefore, the risk score had only demonstrated potential application value in predicting drug treatment outcomes for LUAD patients and required further experimental studies for confirmation.

### Various biomarker-related functional pathways

**Fig. 9:**
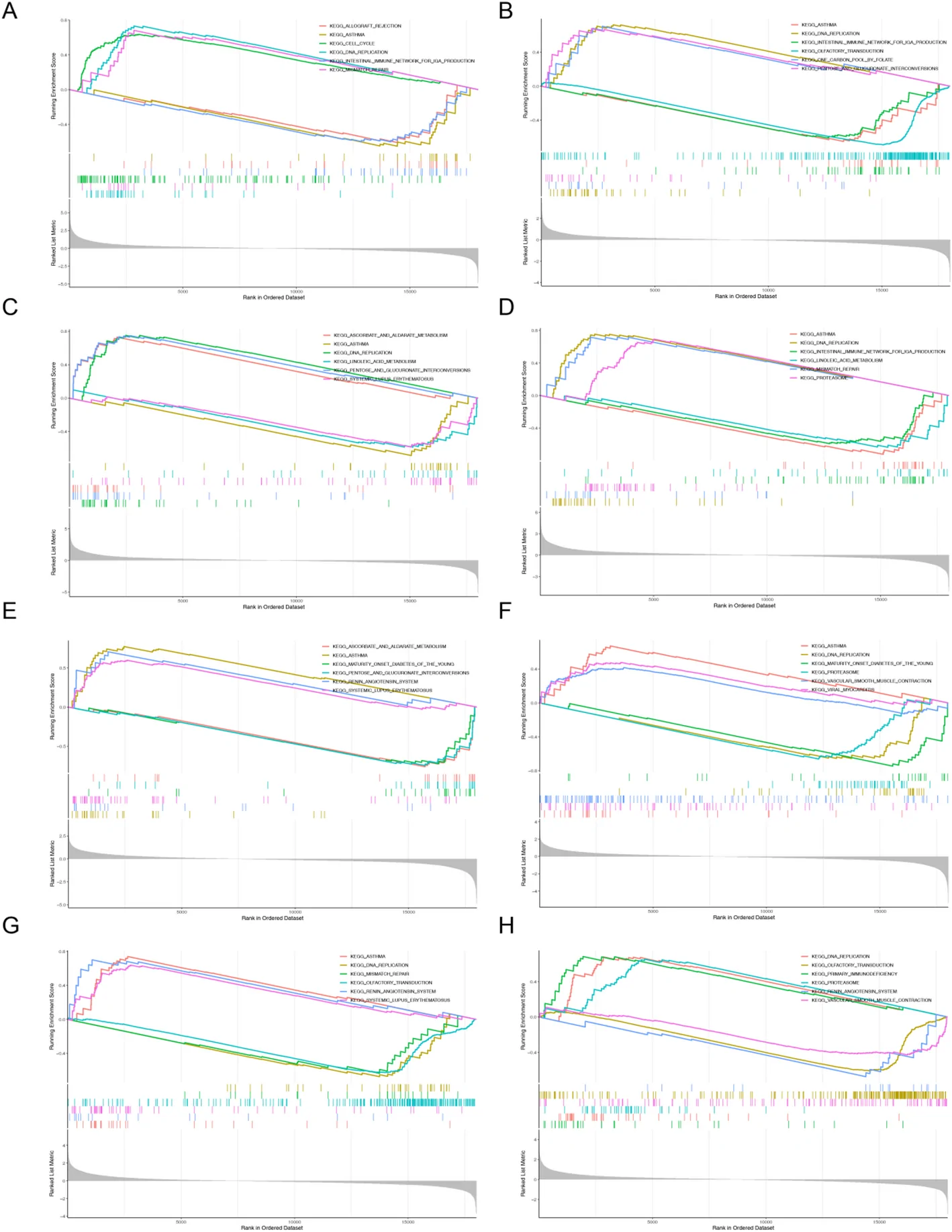
KEGG pathways enriched for genes associated with SLC2A1 (**A**), SMS (**B**), CCNA2 (**C**), CDC25C (**D**), RNASE1 (**E**), NR3C2 (**F**), CAT (**G**) and ADA (**H**). The coloured curves in the figure represent different functional pathways, and the vertical axis represents the enrichment score. The vertical lines in the figure represent genes enriched in the pathways. In the grey area below the figure, the x-axis represents genes sorted by logFC, and the y-axis represents the logFC values

According to the enrichment analysis, SLC2A1, CAT, and CDC25C were mainly associated with mismatch repair and DNA replication. SMS was mainly enriched in intestinal immune network for IGA production, and olfactory transduction. CCNA2 and RNASE1 were mainly involved in ascorbate and aldarate metabolism, pentose and glucuronate interconversions. Furthermore, NR3C2 and ADA mainly participated in vascular smooth muscle contraction, and proteasome (Fig. 9A–H, Supplementary Tables 4–11). Biomarkers might exert critical roles in LUAD prognosis by modulating these critical pathways. Based on the 8 prognostic markers and 11 PANoptosis subtype genes, a protein-protein interaction network was constructed using the STRING database (confidence threshold 0.4). High-confidence interaction pairs were screened and imported into Cytoscape; following the calculation of node connectivity, six hub genes were identified: CYCS, HSPA4, CCNA2, PLK1, CDC25C and FADD (Supplementary Fig. 4A). Reactome enrichment analysis of these six hub genes revealed that 11 of the top 15 significant pathways were associated with cell death, encompassing processes such as apoptosis, pyroptosis and the regulation of necrotic apoptosis (*p* < 0.05) (Supplementary Fig. 4B).

### Expression validation of biomarkers

**Fig. 10:**
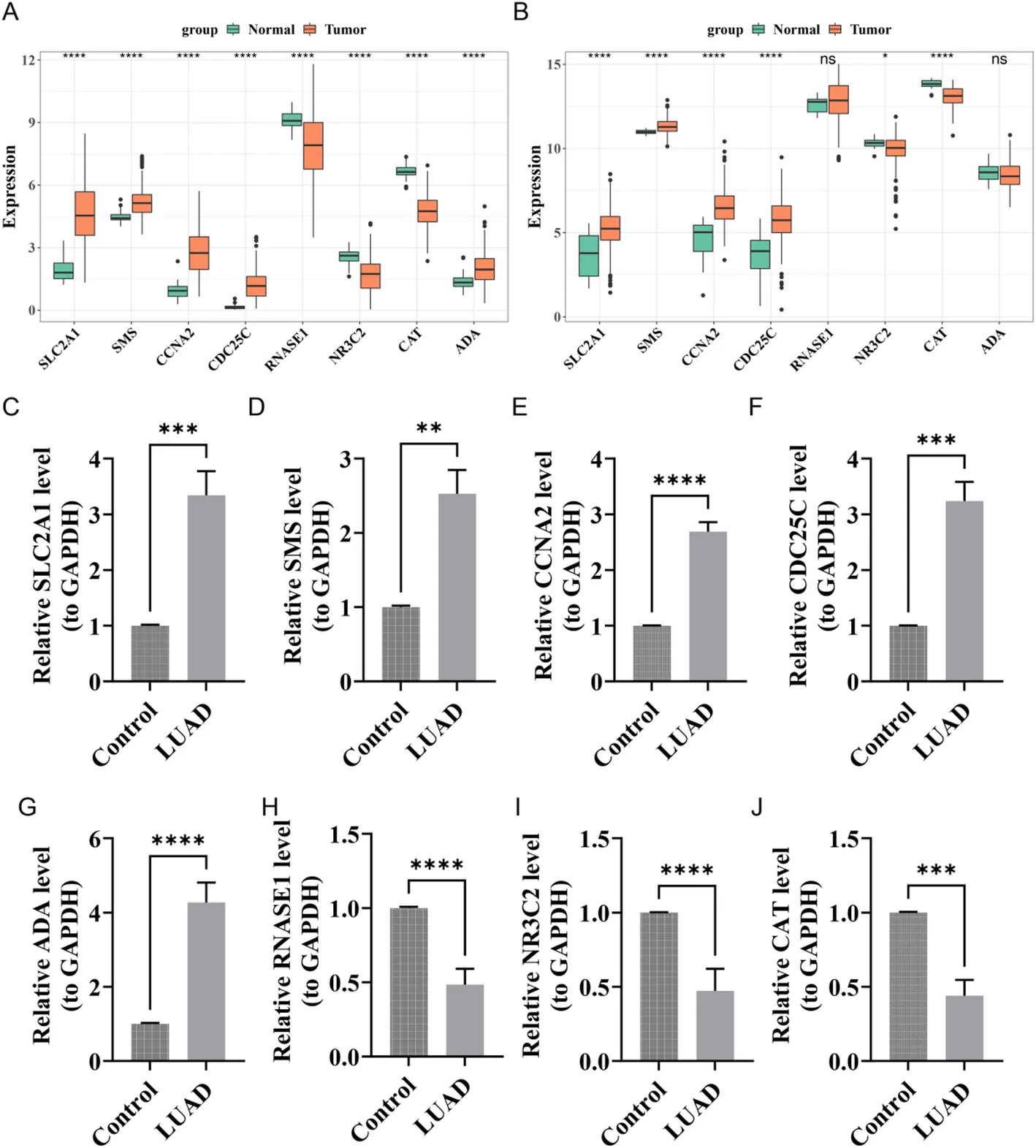
Expression validation of biomarkers. **A** Expression of biomarkers in the training set TCGA-LUAD. **B** Expression of biomarkers in the validation set GSE68465. **C**–**J** Validation of biomarker expression in clinical samples via RT-qPCR. * *p* < 0.05, ** *p* < 0.01, *** *p* < 0.001, **** *p* < 0.0001; ns: not significant

We found that 6 biomarkers including SLC2A1, CAT, SMS, CCNA2, NR3C2, and CDC25C were all remarkably different between LUAD and Normal groups within training and validation sets. Moreover, expression trends across both datasets were consistent. SLC2A1, SMS, CCNA2, and CDC25C were upregulated in LUAD, while NR3C2 and CAT were downregulated (Fig. 10A, B). In clinical samples, the expression of SLC2A1, SMS, CCNA2, CDC25C, and ADA was also markedly increased in LUAD (*p* < 0.01) (Fig. 10C–G), while the expression of RNASE1, NR3C2, and CAT was notably decreased (*p* < 0.001) (Table 3; Fig. 10H–J). This was consistent with the results from the training set. It was worth noting that ADA and RNASE1 did not show significant differential expression in the GSE31210 dataset; this may have been related to differences in sample processing or the detection platform. Their role in LUAD could be further validated by increasing the sample size or conducting functional experiments.

**Table 3:** Validation of biomarker expression in clinical samples via RT-qPCR

	Control	LUAD	FC	*P*-value
SLC2A1	1 ± 0.0388	3.3427 ± 0.9663	3.3427	0.0006
SMS	1 ± 0.0457	2.5276 ± 0.7121	2.5276	0.0014
CCNA2	1 ± 0.0044	2.6896 ± 0.1708	2.6896	< 0.0001
CDC25C	1 ± 0.0122	3.2379 ± 0.7728	3.2379	0.0002
RNASE1	1 ± 0.0091	0.4856 ± 0.1058	0.4856	< 0.0001
NR3C2	1 ± 0.0024	0.4727 ± 0.1481	0.4727	< 0.0001
CAT	1 ± 0.0103	0.4397 ± 0.2373	0.4397	0.0008
ADA	1 ± 0.0257	4.2713 ± 0.5361	4.2713	< 0.0001

## Discussion

Genes implicated in cell death exhibit prognostic relevance in the context of cancer patients [19]. PANapoptosis, a newly characterized modality of cell death, has been correlated by investigators with both cancer cell death and the induction of antitumor immune responses. However, the precise implications of PANapoptosis in the context of LUADs are yet to be comprehensively delineated. Furthermore, with the continuous emergence of more advanced research tools, particularly their applications in gene expression signature analysis and network map construction, an increasing number of small molecules derived from traditional Chinese medicine (TCM) have been identified. As important active components of Cordyceps sinensis, these molecules are widely used in cancer treatment. Furthermore, these applications have promoted the integration of TCM into modern medicine, offering new insights and possibilities for cancer therapy. Cordycepin (CD), as one of the main components of Cordyceps sinensis, has attracted increasing attention from both scientists and clinicians. Previous research has demonstrated that cordycepin can impact the growth of non-small cell lung cancer through various pathways, including influencing its proliferation, migration, and invasion [20]. However, whether CD inhibits the growth of lung cancer cells through the Pan-apoptosis pathway has been rarely investigated. The present study aimed to elucidate the biological effects of cordycepin on LUAD and verify the mechanisms predicted by network pharmacology and Transcriptomics.

In our study, based on network pharmacology analysis, a total of 8 biomarkers including Solute Carrier Family 2 Member 1 (SLC2A1), Spermine Synthase (SMS), Cyclin A2 (CCNA2), Cell Division Cycle 25 C (CDC25C), Ribonuclease A Family Member 1 (RNASE1), Nuclear Receptor Subfamily 3 Group C Member 2 (NR3C2), Catalase (CAT), and Adenosine Deaminase (ADA) were identified through univariate COX regression and LASSO algorithm. Based on the network pharmacology analysis, the potential mechanisms between genes its biomarkers can be effectively elucidated at the system level. Our results demonstrate high expression of SLC2A1 in LUAD, consistent with the pan-cancer analysis reported by Wang et al. [21]. Downregulation of SLC2A1 has been shown to inhibit proliferation, migration and induce apoptosis in non-small cell lung cancer cells [22], supporting its function as a putative oncogene. Several studies have investigated the expression levels of CDC25C protein as a prognostic indicator in tumors. Stern et al. found that well-differentiated LUAD tumors express lower levels of CDC25C, whereas poorly differentiated LUAD tumors express higher levels of CDC25C, thus linking CDC25C expression with aggressive histological features of the tumor [23]. The present study revealed that CDC25C was upregulated in the high-risk group, which further supports the conclusion that high CDC25C expression is associated with invasiveness and poor prognosis in lung adenocarcinoma (LUAD). Alternatively, Chen et al. demonstrated that CAT may modulate the cell cycle and reduce the invasiveness of tumor cells in LUAD by potentially influencing the expression of the P53 gene, thereby impacting the prognosis of LUAD [24].

Although the present study did not directly validate this mechanism, the high expression trend of CAT in the low-risk group suggests that it may act as a protective factor involved in the progression of LUAD. Furthermore, our results indicate that SLC2A1, CDC25C, and CAT are mainly associated with mismatch repair and DNA replication. Previous studies have demonstrated that excessive DNA damage beyond cellular repair capacity can activate the p53-dependent apoptotic pathway and trigger pyroptosis via the caspase-3/GSDME axis [25, 26]. Accordingly, SLC2A1, CDC25C, and CAT may indirectly modulate the sensitivity of LUAD cells to cordycepin-induced PANoptosis by regulating the threshold of DNA damage repair.RNASE1 and CCNA2 were mainly involved in ascorbate and aldarate metabolism, pentose and glucuronate interconversions [27]. RNase1, as an abundant extracellular ribonuclease (ptRNase), plays a role in inflammatory responses and blood coagulation. Our study is the first to reveal a potential association between RNase1 and the prognosis of LUAD, suggesting that it may serve as a prognostic biomarker and provide new insights for LUAD treatment. CCNA2 plays a crucial role in cell cycle regulation, mainly influencing cell proliferation and differentiation through regulating signaling pathways mediated by CDK6 and MET. Studies have reported that in lung cancer, high expression of CCNA2 is significantly associated with adverse clinical features, including longer cycles in the NCCN risk stratification, specific tumor locations, and advanced clinical stages [28]. Moreover, smoking-induced upregulation of CCNA2 can drive the differentiation of AT2/AT2-like cells, thereby contributing to the tumorigenesis of LUAD [29].

The present study further observed significantly elevated expression of CCNA2 in the high-risk subgroup of LUAD, which is consistent with the oncogenic role of CCNA2 reported in previous literature. These findings provide new evidence from a risk stratification perspective supporting the association between CCNA2 and poor prognosis in LUAD.

NR3C2 and ADA mainly participated in vascular smooth muscle contraction, and proteasome [30]. Notably, in the study conducted by Wang et al., NR3C2 has been confirmed as a target gene of miR-31, and its association with overall survival (OS) in LUAD was demonstrated to be inversely correlated [31]. Regarding ADA, in experiments conducted by Chang et al., it was observed that the proliferation of H1299 and PC9 cells was significantly inhibited by knocking out the ADA gene. Subsequent experiments further demonstrated that the expression of ADA significantly promoted the migration and invasion of LUAD cells [32].

In the present study, NR3C2 was upregulated in the low-risk group, while ADA was upregulated in the high-risk group. These expression patterns are highly consistent with the functional roles reported in previous literature, further validating the important prognostic roles of both genes in LUAD.To further explore the immune infiltration landscape of LUAD, the CIBERSORT and ssGSEA algorithms were applied to estimate immune cell infiltration level between the two risk subgroups, respectively. We analyzed the differences in immune cells between high and low-risk groups and identified 10 differential immune cells types (Macrophages M0, Monocytes, Activated Dendritic cells, etc.) between the two subgroups by the CIBERSORT algorithm. Studies have shown that macrophages facilitate the formation of an immunosuppressive microenvironment and impede the infiltration of immune cells, thereby suppressing the activation of anti-tumor immune systems and enabling tumor cells to resist therapy [33]. Furthermore, macrophages can also stimulate tumor angiogenesis and subsequent lung cancer metastasis [34]. Dendritic cells also play an important role in immune responses and have been shown to induce the activation of B cells, thereby enhancing immune activity and influencing the occurrence and development of hepatocellular carcinoma. Our study demonstrated that these immune cells exhibit significant differences in performance between different risk groups, with the immune score and stromal score in the low-risk group being significantly higher than those in the high-risk group. The above results further emphasize the important role of the immune system in tumor control. Meanwhile, the differences in the expression of immune checkpoint genes also support this view. Recent studies have experimentally validated the association between key gene expression, poor prognosis, and immune-related pathways in lung squamous cell carcinoma, further supporting the feasibility of identifying therapeutic targets for lung cancer from the perspective of the immune microenvironment [35]. Accordingly, future research may focus on exploring strategies to modulate the immune microenvironment to improve prognosis in LUAD patients. For instance, targeting the reprogramming of M2-type macrophages has been shown to synergistically enhance anti-super immune responses [36], providing potential targets for immunotherapy of LUAD.

Among the drugs involved in this study, cisplatin, a classic chemotherapeutic agent, has been widely used as first-line therapy for lung cancer and other solid tumors. It exerts cytotoxic effects by disrupting tumor cell DNA, though drug resistance remains a major hurdle in clinical applications [37]. Parthenolide, a natural compound, possesses anti-inflammatory activity and can induce tumor cell apoptosis; preclinical studies in lung cancer, liver cancer, and various other tumors have confirmed its capacity to enhance the efficacy of chemotherapy or immunotherapy [38].

Drug sensitivity analysis in this study identified significant differences in the IC50 values of 22 drugs between high-risk and low-risk groups. Among these agents, AS601245, CCT007093, and DMOG exhibited a positive correlation with risk scores (correlation coefficient > 0.3), whereas cisplatin, CMK, and parthenolide showed a negative correlation (correlation coefficient < − 0.3). These findings suggest that patients stratified by different risk levels display distinct responses to therapeutic drugs. The biomarker-based risk model established herein reveals the associations of lung adenocarcinoma (LUAD) prognosis with the tumor immune microenvironment and drug sensitivity, thereby providing a theoretical basis for individualized treatment strategies. In the future, representative drugs (such as cisplatin or parthenolide) may be selected to conduct CCK-8 proliferation inhibition assays, colony formation assays, and IC₅₀ determinations in LUAD cell lines, so as to verify the actual differences in drug sensitivity among different risk subgroups.

Furthermore, gene interventions (such as knockdown or overexpression of key biomarkers) will be further combined to explore the mechanisms by which risk model–related genes affect drug responses, thereby providing experimental evidence for individualized treatment strategies based on this risk score.

GSEA further linked our identified biomarkers to key biological pathways: SLC2A1, CAT, and CDC25C were primarily enriched in mismatch repair and DNA replication; SMS in the intestinal immune network for IgA production and olfactory transduction; CCNA2 and RNASE1 in ascorbate/aldarate metabolism and pentose-glucuronate interconversions and NR3C2 and ADA in vascular smooth muscle contraction and the proteasome pathway. These pathways are well-documented in tumorigenesis and progression, contextualizing the biomarkers’ functional role in LUAD. Mismatch repair and DNA replication, for instance, are critical for genomic stability, and their dysregulation is associated with carcinogenesis, chemotherapy resistance (e.g., to platinum agents), and poor prognosis in LUAD and other malignancies [39]. The intestinal immune network linked to SMS participates in mucosal immunity, with its dysregulation potentially impairing antitumor surveillance—particularly relevant in lung cancers with immune microenvironment dependencies. Metabolic pathways such as ascorbate/aldarate metabolism, enriched for CCNA2 and RNASE1, underpin tumor metabolic reprogramming, supporting rapid proliferation by supplying energy and biosynthetic precursors, and their targeting has emerged as a promising therapeutic strategy in LUAD [40]. Meanwhile, vascular smooth muscle contraction (associated with NR3C2) intersects tumor angiogenesis and metastasis [41], as abnormal vascular regulation facilitates tumor cell extravasation, and the proteasome pathway (linked to ADA) regulates cell survival and resistance to proteasome inhibitors in solid tumors. Collectively, these findings clarify how our 8 biomarkers influence LUAD progression through interconnected pathways spanning genomic stability, metabolism, immunity, and vascular biology, deepening understanding of the molecular crosstalk driving LUAD. Clinically, this supports targeted interventions: e.g., inhibiting CDC25C-associated mismatch repair could sensitize LUAD to DNA-damaging agents, modulating CCNA2-linked metabolic might disrupt tumor metabolic adaptation; combining immune checkpoint inhibitors with IgA-related modulators could enhance antitumor immunity. These pathways and biomarkers may also serve as prognostic indicators, aiding patient stratification for personalized LUAD therapy.

In this study, through integrated network pharmacology and transcriptomic analyses, we identified 8 key genes with significant prognostic value in LUAD, and a risk model with robust predictive performance was constructed. Our research, however, still has some limitations. Several limitations should be acknowledged in the present study. To begin with, the overall sample size is relatively limited and mainly obtained from public databases, which may lead to selection bias. In addition, the small sample size of clinical validation (*n* = 5) limits statistical power and makes it difficult to assess population heterogeneity and perform subgroup analyses. Furthermore, this study only identified a statistical correlation between the eight genes and the prognosis of LUAD without functional experimental verification. Only mRNA expression levels were detected without validation at the protein level. Therefore, the current conclusions mainly reflect the correlation between gene expression and prognosis, and the direct mechanism underlying their roles in the occurrence and progression of LUAD remains to be clarified.

Notably, although static network methods including protein–protein interaction (PPI) analysis have been widely applied to screen potential biomarkers in cancer research, they fail to capture the temporal dynamic characteristics of molecular interactions [42]. Moreover, despite the eight biomarkers being derived from the PANoptosis-related gene set, there is a lack of direct experimental evidence linking them to the PANoptosis process in LUAD. Thus, the speculation regarding their involvement in PANoptosis is indirect and should be interpreted with caution. In addition, this study mainly relied on transcriptomic data without integrating multi-omics information such as epigenomic data, whereas the anti-tumor mechanism of cordycepin may involve epigenetic regulation including DNA methylation and histone modification. Future studies are warranted to validate the robustness of the model in larger-scale and multi-center cohorts, and expand the clinical sample size to confirm the expression heterogeneity of key genes.

Meanwhile, loss-of-function or gain-of-function strategies such as CRISPR-Cas9 screening should be adopted for causal verification, combined with proteomic analysis to clarify gene functions [43, 44].

Further determination of the IC50 value of cordycepin in LUAD cell lines is needed. After stratification based on the expression levels of biomarkers (e.g., SLC2A1), treatment with cordycepin alone or combined with siRNA intervention targeting key genes can be performed. The combination index (CI) should be calculated using the Chou-Talalay method to quantitatively evaluate the synergistic effect between targeted genes and cordycepin [45].

Additionally, dynamic network modeling should be integrated to reveal time-dependent regulatory mechanisms [46].

More importantly, future multi-omics studies including whole-genome DNA methylation sequencing (WGBS), ChIP-seq and ATAC-seq should be conducted to construct a regulatory network of “cordycepin-epigenetics-transcriptome-phenotype” and further elaborate the complete molecular mechanism by which cordycepin treats LUAD through the PANoptosis pathway.

## Conclusion

This study integrated network pharmacology and transcriptomic analyses to systematically explore the prognostic significance of PANoptosis-associated genes in lung adenocarcinoma (LUAD), as well as their latent correlation with cordycepin-mediated anti-tumor therapy. From the PANoptosis-related gene set, we successfully screened and identified eight pivotal prognostic biomarkers consisting of SLC2A1, SMS, CCNA2, CDC25C, RNASE1, NR3C2, CAT, and ADA, and constructed a risk scoring model for predicting the prognosis of LUAD. Notably, this established model is capable of effectively stratifying LUAD patients into distinct risk subgroups based on their prognosis, and it exhibits significant correlations with the tumor immune microenvironment landscape and sensitivity to multiple chemotherapeutic or targeted agents. Accordingly, our findings not only provide novel and promising candidate biomarkers for clinical prognostic evaluation of LUAD but also offer a preliminary theoretical foundation for the development of individualized and precise therapeutic strategies targeting this malignancy.

Nevertheless, it should be emphasized that the current conclusions are derived from bioinformatic analyses and retrospective data mining, and the corresponding results remain to be further validated. Future large-scale, multi-center prospective cohort studies are urgently needed to verify the stability and clinical applicability of this risk score model. Additionally, in-depth functional experiments, particularly causal validation techniques including CRISPR-Cas9 gene editing and loss- or gain-of-function assays, are warranted to elucidate the specific molecular mechanisms underlying the roles of these eight key genes in PANoptosis regulation during LUAD initiation and progression, as well as their precise interaction with cordycepin in anti-tumor responses.

## Data Availability

The datasets generated and/or analyzed during the current study are available in publicly accessible repositories. The TCGA-LUAD data and clinical information can be accessed via the UCSC Xena browser (https://xenabrowser.net). The gene expression microarray datasets GSE68465 and GSE31210 are available in the NCBI Gene Expression Omnibus (GEO) database (https://www.ncbi.nlm.nih.gov/geo/). The target gene data were retrieved from publicly available databases, including TCMSP (https://old.tcmsp-e.com/tcmsp.php), SYMMAP (http://www.symmap.org), CTD (http://ctdbase.org/), Super-pred (https://prediction.charite.de/), SEA (https://sea.bkslab.org/), and HERB (http://herb.ac.cn).
